# *MLH1* Constitutional Epimutation Screening Requires Highly Sensitive Assays to Identify Lynch Syndrome Patients With Very Low Mosaic Methylation Level

**DOI:** 10.1155/humu/6909313

**Published:** 2026-05-14

**Authors:** Cédric Facon, Catherine Vermaut, Lucie Delattre, Cathy Flament, Antoine Dardenne, Afane Brahimi, Sophie Lejeune, Stéphane Cattan, Francoise Bonnet, Noémie Basset, Anika Bensen, Anais Dupré, Pascaline Berthet, Marion Dhooge, Chrystelle Colas, Emmanuelle Mouret-Fourme, Hélène Delhomelle, Sophie Nambot, Amandine Baurand, Marc Planes, Julie Menjard, Patrick R. Benusiglio, Pascal Pigny, Marie-Pierre Buisine, Julie Leclerc

**Affiliations:** ^1^ EpiCARe,Team Univ. Lille, Inserm, CHU Lille, CNRS, Centre Oscar Lambret, U1366-UMR9020, CRCLille (Cancer Research Center of Lille), Lille, France, chru-lille.fr; ^2^ Molecular Oncogenetics Unit, Department of Biochemistry and Molecular Biology, Lille University Hospital, Lille, France, chru-lille.fr; ^3^ General and Digestive Surgery, Saint-Antoine Hospital, APHP, Sorbonne University, Paris, France, sorbonne-universites.fr; ^4^ Clinical Genetics Department, Lille University Hospital, Lille, France, chru-lille.fr; ^5^ Digestive Oncology Unit, Lille University Hospital, Lille, France, chru-lille.fr; ^6^ Cancer Genetics Department, Institut Bergonié, Bordeaux, France; ^7^ Department of Medical Genetics, Pitié-Salpêtrière Hospital, APHP, Sorbonne University, Paris, France, sorbonne-universites.fr; ^8^ Oncogenetics Department, Centre François Baclesse, Caen, France; ^9^ Gastroenterology Unit, Cochin Teaching Hospital, APHP Centre Université de Paris, Paris, France; ^10^ Genetics Department, Institut Curie, PSL Research University, Paris, France, univ-psl.fr; ^11^ Inserm U830, DNA Repair and Uveal Melanoma (D.R.U.M.), Paris, France; ^12^ Oncogenetics Unit, Centre de Lutte Contre le Cancer Georges François Leclerc-UNICANCER, Dijon, France; ^13^ Genetics Center, FHU-TRANSLAD, Dijon-Bourgogne University Hospital, Dijon, France; ^14^ Genetics Department, Brest University Hospital, Morvan Hospital, Brest, France

**Keywords:** Lynch syndrome, *MLH1* constitutional epimutation, *MLH1*-methylated tumors, mosaic promoter methylation, oncogenetics

## Abstract

Constitutional epimutations of the *MLH1* gene are an alternative cause of Lynch syndrome, in which inactivation of an allele of a mismatch repair (MMR) gene results from *MLH1* promoter methylation, rather than a pathogenic genetic variant. These epimutations are often mosaic, and methylation levels ranging from ~50% monoallelic methylation to low‐level methylation (1%–5%) are observed in the blood of *MLH1* epimutation carriers. Using a specific and highly sensitive droplet digital methyl‐specific PCR (ddMSP) assay, six patients with very low methylation levels (< 1%) were identified in a series of 142 patients with a *MLH1*‐methylated tumor diagnosed before age 61, who had been referred to the clinical lab between 2020 and 2024. These patients were initially missed by standard pyrosequencing assay, emphasizing the need for highly sensitive assays for constitutional epimutation screening. To confirm that methylated DNA molecules detected by ddMSP did not correspond to circulating tumor DNA rather than germline DNA, multiple validation analyses were performed, including validation of the constitutional origin of methylation on other sources of germline DNA and tumoral analysis. Taking into account the other patients identified as epimutation carriers by pyrosequencing during the same 5‐year period, 13.1% of patients with a *MLH1*‐methylated tumor before age 61 were diagnosed as Lynch syndrome patients, which changed their clinical follow‐up. These findings highlight the relevance of recommendations for systematic *MLH1* epimutation screening using highly sensitive assays in patients with *MLH1*‐methylated tumors diagnosed before age 61. Such screening will increase the number of patients diagnosed with Lynch syndrome caused by a *MLH1* constitutional epimutation, improving patient care and outcomes, as well as genetic counseling.

## 1. Introduction

Lynch syndrome (LS) is an autosomal dominant hereditary condition predisposing to cancer, mainly of the colon, rectum, and endometrium [1, 2]. It is caused by a constitutional variant inactivating one allele of a gene, which encodes a protein involved in DNA mismatch repair (MMR): *MLH1*, *MSH2*, *MSH6*, or *PMS2*. Inactivation of the second allele of the same gene in somatic cells leads to mismatch repair deficiency (dMMR) and subsequent accumulation of replication errors, promoting tumorigenesis. MMR deficiency is a hallmark of LS‐associated tumors that can be detected using immunohistochemistry (IHC) to look for loss of MMR protein expression and microsatellite instability (MSI), which corresponds to the accumulation of replication errors in repetitive microsatellite sequences and is a consequence of dMMR.

Most LS patients carry a constitutional genetic variant in a MMR gene. However, for some patients, the constitutional alteration that inactivates one allele of a MMR gene and is responsible for LS is epigenetic [3, 4]. These patients have a constitutional epimutation, that corresponds to monoallelic methylation of the promoter CpG island leading to transcriptional repression and gene silencing, throughout normal tissues. Constitutional epimutations have been reported in *MLH1* [5] and *MSH2* [6] genes. *MSH2* constitutional epimutations are associated with deletions of variable size affecting the 3 ^′^ region of the *EPCAM* gene (located upstream of the *MSH2* gene) which are responsible for the expression of a fusion transcript and *MSH2* promoter methylation [7]. As this *MSH2* epigenetic silencing is observed in *EPCAM*‐expressing tissues, patients have a modified LS phenotype with mainly high risk for colorectal cancers [8]. Molecular mechanisms behind *MLH1* constitutional epimutations are more diverse. Some of them arise *de novo* and correspond to pure epigenetic events, labile in the germline. These epimutations, named primary, are typically not transmitted to the offspring, although occasional non‐Mendelian inheritance has been observed [9, 10]. *MLH1* epimutations can also be linked to a *cis*‐acting genetic alteration [11–16]. These epimutations, named secondary, are thus transmitted to the offspring following a Mendelian autosomal dominant inheritance pattern. Secondary epimutations appear to be less frequent than primary epimutations [17].

Identification of patients carrying a *MLH1* constitutional epimutation is hampered because of *MLH1* methylation observed in their tumors. As biallelic methylation of *MLH1* promoter is a frequent mechanism leading to sporadic dMMR tumors, methylation analysis in tumors exhibiting loss of MLH1 expression is used in current guidelines to select patients who should be referred to a geneticist for germline genetic testing [1]. Tumors that are *MLH1*‐methylated are considered sporadic, with no germline genetic testing required, whereas tumors that are unmethylated on *MLH1* promoter are considered to be potentially LS‐associated, warranting germline genetic testing. Consequently, epimutation carriers are too often considered as having a sporadic tumor because of *MLH1* methylation, and excluded from further germline testing and epimutation detection. However, these patients need to be identified as they require LS‐dedicated clinical follow up.

Variable levels of methylation are observed in the blood of *MLH1* epimutation carriers [17]. Methylation can be hemiallelic, with all PBMC (peripheral blood mononuclear cells) exhibiting monoallelic methylation of the *MLH1* promoter. In these cases, blood‐based methylation level is ~50%. Constitutional epimutations are also frequently mosaic, with some PBMC exhibiting monoallelic methylation and other PBMC exhibiting two unmethylated *MLH1* alleles. In these cases, methylation levels < 40%–50% are detected, and low‐level (1%–5%) can be observed for some patients [16, 18–20].

Pyrosequencing, which is commonly used in clinical labs for promoter methylation analysis of genes such as *MGMT* [21] and *MLH1* in tumors, is also widely used for constitutional epimutation screening since this technique has a higher analytical sensitivity than MS‐MLPA (~3%–5% versus ~10%). However, the background signal observed on pyrograms makes detection of low methylation levels difficult, as they are hard to distinguish from a negative sample. Techniques with higher sensitivity have been developed and are slowly replacing pyrosequencing. They are based on real‐time PCR (quantitative PCR [qPCR]), melting curve analysis (MCA) or digital PCR (dPCR). They can be applied to methylation detection, in a methyl‐specific (MS) version: qMSP (quantitative methyl‐specific PCR) [19, 20], MS‐MCA [18] or digital MS PCR [22].

As we implemented a more sensitive droplet digital methyl‐specific PCR (ddMSP) assay in the clinical lab, we decided to retest blood‐extracted DNA samples previously negative with pyrosequencing and identified patients with very low level of constitutional *MLH1* methylation.

## 2. Patients and Methods

### 2.1. Patients

One hundred forty‐six patients were included in this study. These patients had been referred to the lab by geneticists from University Hospitals and Cancer Treatment Centers throughout France between 2020 and 2024 for *MLH1* epimutation screening (first‐line molecular testing or after negative *MLH1* mutational screening) because they developed a dMMR tumor (with loss of MLH1 and PMS2 protein expression by IHC and/or MSI) with somatic hypermethylation of *MLH1* promoter. Methylation analysis had been performed on blood‐extracted DNA using pyrosequencing and was negative. These patients were selected for reanalysis with a higher‐sensitivity technique because the age of tumor diagnosis was below 61 years old. Due to the lack of remaining DNA samples, reanalysis could not be performed for three patients, resulting in 143 patients with DNA samples available for reanalysis. Written informed consent had been obtained from all individuals.

Over the same period (2020–2024), 15 patients with a *MLH1*‐methylated tumor ≤ 60 years old were identified as *MLH1* constitutional epimutation carriers using pyrosequencing. These patients are not further detailed as this study focused on patients with negative pyrosequencing testing, but they were used for frequency calculation. Two of these patients, with low‐level constitutional *MLH1* methylation detected by pyrosequencing, are however detailed in order to highlight the relevance of ddMSP as an alternative highly sensitive technique to validate pyrosequencing results and to compare methylation levels determined by these two techniques.

### 2.2. Samples

DNA had been extracted from blood using the Chemagic STAR Blood 1K kit (Perkin Elmer) for 109 patients (76%, 109/143). For 34 patients (24%, 34/143), blood‐extracted DNA had been transmitted to the lab for epimutation screening. Additional samples were requested for the validation of very low methylation levels, including independent blood samples and samples originating from other embryonic layers. Non‐opposition of the corresponding patients was recorded upon their inclusion in a biobank and research project approved by an independent Ethical Research Committee (Comité de Protection des Personnes Sud‐Est 1, 2021‐047), in compliance with French regulations on research involving human subjects (RIPH3). DNA was extracted from 1 mL of blood (collected on EDTA tubes), 1 mL of saliva (collected on Oragene tubes from DNA Genotek), 1 mL of H_2_O in which a buccal swab incubated overnight at 56°C or of buccal swab preservation solution, using the Chemagic STAR Blood 1K kit. After deparaffinization (deparaffinization solution, Qiagen), DNA was extracted from FFPE (formalin‐fixed paraffin‐embedded) tumor samples or from FFPE non‐tumoral tissue samples using the QIAamp DNA FFPE Tissue kit (Qiagen). To increase the ratio of DNA coming from tumoral cells, tumor samples were first macrodissected based on the demarcation by a pathologist of the area of high tumor cellularity on a hematoxylin and eosin‐stained slide. The percentage of MSI cells in the sample was evaluated based on the profile of the MSI test. Non‐tumoral tissue distant from the tumor was obtained from a different paraffin block, labeled as non‐tumoral.

The cell‐free DNA ScreenTape assay and the 4200 TapeStation system (Agilent) were used to look for circulating DNA (size 50–800 bp) in DNA samples extracted from blood.

### 2.3. *MLH1* Promoter Methylation Analysis

DNA (100–250 ng for blood‐extracted DNA, available amount up to 100 ng for tissue‐extracted DNA) was treated with sodium bisulfite using the EZ DNA Methylation kit (Zymo Research), according to the manufacturer′s instructions. Elution was performed with 10 *μ*L M‐Elution Buffer.

**Figure 1:**
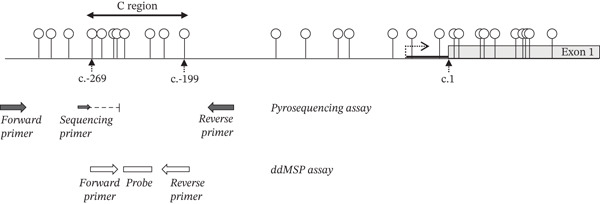
Map of the CpG at the 5 ^′^ end of *MLH1* gene, with location of the pyrosequencing and ddPCR assays. Each lollipop represents the C of an individual CpG. The CpG included in the C region described by Deng et al. [23] are indicated, with nucleotide position of the first and the last C in the reference sequence (NM_000249.4). The transcriptional start site is indicated by a dotted arrow. The c.1 nucleotide is the A of the translational initiation codon. Pyrosequencing primer positions are depicted as black arrows, the thinner one corresponding to the sequencing primer position. The dotted line represents the region that is sequenced (from nucleotide c.−269 to nucleotide c.−249), including 4 CpG sites (at positions c.−269, c.−262, c.−252 and c.−250). ddMSP primer positions are depicted as white arrows (nucleotide c.−270 to nucleotide c.−250 for the forward primer and nucleotide c.−215 to c.−195 for the reverse primer), and the probe position is depicted as a white rectangle (nucleotide c.−246 to nucleotide c.−224).

The pyrosequencing assay is based on the PyroMark *MLH1* kit (Qiagen) which targets four consecutive CpG sites within the C‐region described by Deng et al. [23] in *MLH1* promoter (Figure 1). Methylation of this region is strongly correlated with transcriptional silencing [23]. PCR amplification was performed in a final volume of 25 *μ*L containing 0.2 *μ*M of each primer, 1X Multiplex PCR Master Mix (Qiagen), and 15 ng of bisulfite‐treated DNA. Following a denaturation step at 95°C for 15 min, 45 cycles of denaturation at 95°C for 20 s, annealing at 55°C for 20 s, and extension at 72°C for 20 s were carried out, followed by a final extension at 72°C for 5 min. Eight to 12 *μ*L of the PCR product were submitted to pyrosequencing on a PyroMark Q24 system (Qiagen), according to the manufacturer′s instructions. The ratio of cytosines to thymines for targeted CpG sites, which corresponds to the ratio of methylated to unmethylated cytosines, was determined by the PyroMark Q24 software (Qiagen). The mean methylation level is calculated based on the last two CpG sites.

The ddMSP assay is based on two sets of primers and probe: one for the specific amplification and detection of methylated templates from bisulfite‐treated DNA (FAM‐labeled probe) and one for the specific amplification and detection of unmethylated templates (HEX‐labeled probe). Locations of the primers and the probe are the same in these two sets. The primers are located within the Deng‐C region of *MLH1* promoter and enable amplification from c.−270 to c.−195 (NM_000249.4) (Figure 1). The probes hybridize on 2 CpG sites (nucleotide c.−244 and c.−225). Primer and probe sequences are provided in Table S1. The set of primers and probe used for methylated templates has been previously described by Pinto et al. [22]. These sets of primers and probe were chosen because of their specificity in discriminating between methylated and unmethylated DNA templates (Figure S1). Linearity of quantification was also checked (Figure S2).

Droplet dPCR was performed in a total volume of 20 *μ*L with 900 nM primer/250 nM probe for the unmethylated‐specific set of primers and probe and 450 nM primer/125 nM probe for the methylated‐specific set of primers and probe, 10 *μ*L of ddPCR Supermix for Probes (no dUTP) 2X (BioRad) and up to 90 ng of bisulfite‐converted DNA. Droplets were generated using the QX200 Automated Droplet Generator (BioRad). PCR amplification was then performed within the droplets using the following conditions: 95°C for 10 min followed by 40 cycles of 94°C for 30 s and 53.5°C for 1 min, and a final step of 98°C for 10 min. End‐point fluorescence signals were measured by the QX200 Droplet Reader and analyzed by QuantaSoft Analysis software v1.6.6.0320 (BioRad). The total number of DNA molecules analyzed per sample was determined. A minimum of 3000 DNA molecules screened for each sample was set to warranty detection of 1 positive molecule out of 1000 (0.1%) with good confidence (Figure S3). Fractional abundance, corresponding to the methylation level, was determined for each sample using Poisson statistical distribution law with 95% confidence intervals.

The HCT116 CRC cell line was used as *MLH1*‐unmethylated control. The RKO CRC cell line, which exhibits biallelic *MLH1* methylation, was diluted to 50% methylation (RKO 50%), 0.25% methylation (RKO 0.25%), or 0.1% methylation (RKO 0.1%) with DNA from HCT116 and used as *MLH1*‐methylated controls.

### 2.4. Loss of Heterozygosity (LOH) Detection in Tumors

LOH was determined by assessing allelic imbalance of seven SNPs located in the *MLH1* gene (rs1800734, rs9852378, rs4647215, rs3774341, rs9855475, rs4647277, and rs6772548) using pyrosequencing. Allelic ratios were compared between tumoral tissue and non‐tumoral tissue for informative SNPs (i.e., heterozygous in non‐tumoral tissue). Blood was used as the reference when non‐tumoral tissue was not available (Patient B). PCR‐amplification was performed on 25 ng of DNA, using the same protocol as the one used for *MLH1* methylation analysis except for the annealing temperature, which was 58°C. Pyrosequencing was performed with the PyroMark Q24 system (Qiagen) and allelic ratios were determined using the PyroMark Q24 software.

### 2.5. Germline *MLH1* Variant Screening

The non‐coding region around *MLH1* exon 1 (from nucleotide c.−204 to c.116+195) was analyzed using Sanger sequencing. Primer sequences are provided in Table S1. PCR‐amplification was performed in a final volume of 25 *μ*L containing 0.75 U of AmpliTaq Gold (ThermoFisher scientific), 0.3 *μ*M of each primer, 1 mM of dNTPs, 2.5 mM of MgCl2, 1X PCR buffer II and 50 ng of DNA. The PCR conditions were as follows: initial denaturation step at 95°C for 7 min, followed by 40 cycles of denaturation at 95°C for 30 s, annealing at 60°C for 30 s, and extension at 72°C for 1 min, and a final extension step at 72°C for 10 min. Sequencing was performed using BigDye Terminator v3.1 Cycle Sequencing kit (ThermoFisher) on PCR products purified with ExoSAP‐IT PCR Product Cleanup (ThermoFisher Scientific), and a 3730 DNA Analyzer (Applied Biosystems). The SeqScape software v4.0 (Applied Biosystems) was used for sequence data analysis.

## 3. Results

### 3.1. Identification of Patients With Very Low Level of Constitutional *MLH1* Methylation

**Figure 2:**
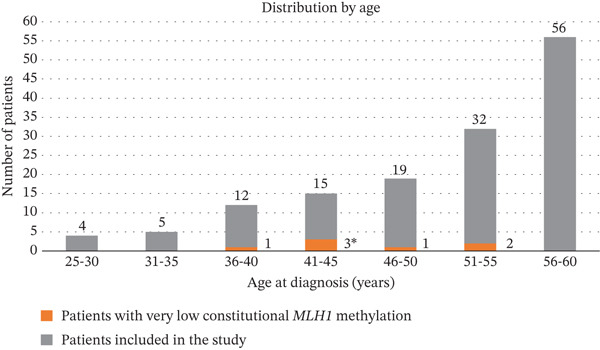
Diagnosis of patients with very low level of constitutional *MLH1* promoter methylation. Histogram showing the number of patients with very low constitutional *MLH1* methylation (in orange, numbers indicated on the right of the histogram bars) detected among cancer cases with a dMMR *MLH1*‐methylated tumor diagnosed ≤ 60 years old who were previously considered as nonepimutation‐carriers with pyrosequencing (in grey, numbers indicated above the histogram bars). Five‐year age bins are represented. ∗As no control sample was available for validation, one patient was excluded from further analyses.

One hundred forty‐six patients who presented with a *MLH1*‐methylated cancer before the age of 61 and were tested negative for constitutional epimutation by pyrosequencing between 2020 and 2024 were included in this study. A DNA sample was available for reanalysis for 143 patients. Among these 143 patients, 107 patients were female and 36 were male. Median age at first tumor diagnosis was 54 years old (range 25–60 years old). Distribution by age range is presented in Figure 2 and Figure S4. Ninety‐two patients (56 female and 36 male) had a digestive cancer (77 colorectal cancers, 6 small intestine cancers, 6 gastric cancers, 1 esophageal cancer, 1 pancreatic cancer, and 1 ampullary cancer) and 51 patients had a gynecological cancer (49 endometrial cancers, 1 ovarian cancer and 1 endometrial cancer with synchronous ovarian cancer).

**Figure 3:**
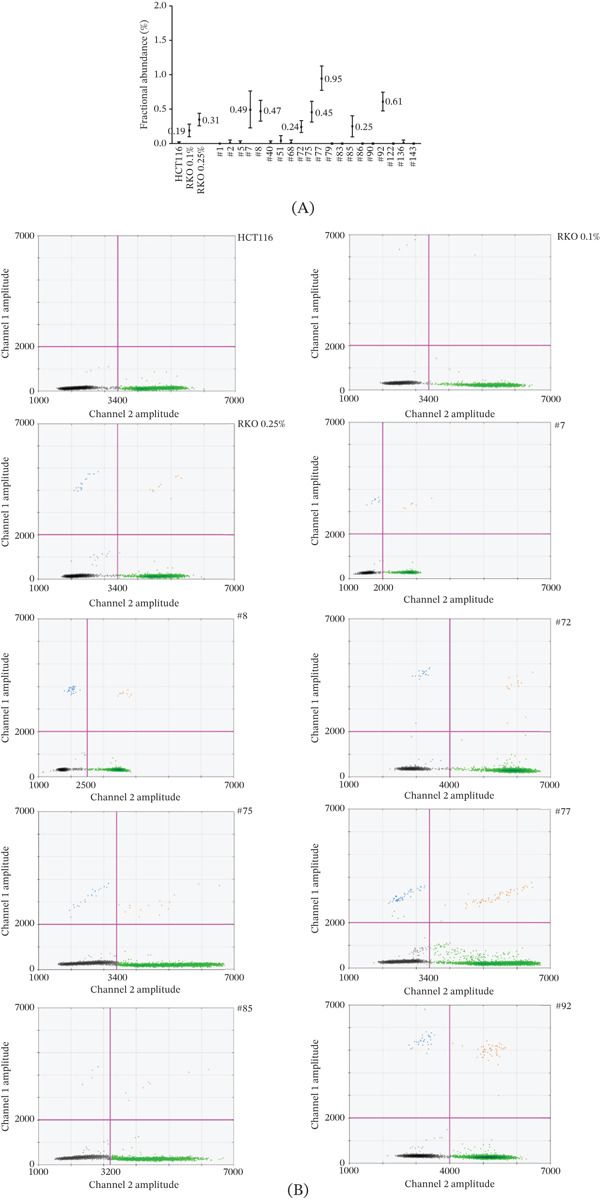
Analysis of *MLH1* methylation level in a series of 143 patients with a dMMR *MLH1*‐methylated tumor diagnosed ≤ 60 y.o., and previously considered as nonepimutation‐carriers with pyrosequencing. (A) *MLH1* methylation levels assessed by ddMSP in bisulfite‐converted DNA from the *MLH1*‐unmethylated HCT116 CRC cell line (pooled results from triplicates with independent bisulfite conversion), from the *MLH1*‐methylated RKO CRC cell line diluted to 0.1% methylation (RKO 0.1%) or to 0.25% methylation (RKO 0.25%) with DNA from HCT116 (pooled results from triplicates with independent bisulfite conversion) and from PBMC of the positive patients and of a few representative negative patients. Fractional abundance, corresponding to the methylation level, was determined for each sample using Poisson statistical distribution law, with errors bars indicating the minimum and maximum values as calculated with Poisson law. (B) ddMSP profiles of HCT116, RKO 0.1%, RKO 0.25% and of the 7 positive patients (#7, #8, #72, #75, #77, #85, and #92). Fluorescence intensity of the individual droplets. FAM‐positive droplets (blue) correspond to amplification of methylated DNA. HEX‐positive droplets (green) correspond to amplification of unmethylated DNA. Red droplets correspond to amplification of both methylated and unmethylated DNA in the same droplet. Grey droplets correspond to empty droplets (no amplification).

Using ddMSP, a very low level of methylation (ranging from 0.24% to 0.95%) was detected in the DNA extracted from blood of 7 patients (Figure 3). A median of 11,620 DNA molecules (range 3840–24,200) were screened per sample, providing strong confidence in negative results. Clinical and pathological data of patients with very low *MLH1* methylation level are summarized in Table 1. Median age at first tumor diagnosis was 45 years old (range 36–55 years old) (Figure 2). Five patients developed a proximal colon cancer, one patient a duodenal cancer and one patient an endometrial cancer. Patient #7 also developed a metachronous endometrial adenocarcinoma 2 years after initial colon cancer. None of the patients had a family history fulfilling Amsterdam criteria for LS.

**Table 1:** Clinical and pathological data of the seven patients with very low *MLH1* methylation level.

Patient	Gender	Tumor	Age at diagnosis	MSI status	MMR protein expression (IHC)	*BRAF* V600E	Family history	Epimutation screening in relatives
#7	F	Proximal colon adenocarcinoma pT4bNO/endometrial endometrioid adenocarcinoma	48/50	MSI	MLH1 and PMS2 loss	WT/NA	2nd degree relative: 1 CRC (75)	Not performed
#8	M	Proximal colon adenocarcinoma with micropapillary component	54	MSI	MLH1 and PMS2 loss (+MSH6 loss in micropapillary component only)	WT	1st degree relative: 1 CRC (60); 2nd degree relative: 1 CRC (76)	2 sons (age < 25 years old): negative^b^
#72^a^	F	Proximal colon adenocarcinoma (ileocecal valve)	42	MSI	MLH1 and PMS2 loss	WT	1st degree relative: 1 CRC (> 90); 2nd degree relatives: 1 EC (55) + 1OC (56), 1 CRC (> 90)	Not performed
#75	F	Proximal colon adenocarcinoma pT3NO	36	MSI	MLH1 and PMS2 loss	WT	1st degree relative: 1 CRC (50) pMMR; 2nd degree relative: 1 CRC (70); 3rd degree relative: 1 CRC (40)	Sister: negative (pMMR CRC 50 years old)
#77	M	Proximal colon adenocarcinoma pT3NO (transverse colon)	45	MSI	MLH1 and PMS2 loss	WT	2nd degree relative: 1 anal cancer	Not performed
#85	F	Endometrial endometrioid adenocarcinoma (FIGO stage 1A, histologic grade II)	41	MSI	MLH1 and PMS2 loss	NA	None	Not performed
#92	M	Duodenal adenocarcinoma	55	MSI	MLH1 and PMS2 loss	WT	1st degree relative: 1 esophageal cancer (87)	Not performed

Abbreviations: CRC, colorectal cancer; F, female; M, male; MSI, microsatellite instability; NA, not applicable (endometrium); WT, wild‐type.

^a^As no control sample was available for validation, this patient was excluded from further analyses.

^b^PCR‐HRM MethylDetect kit.

### 3.2. Validation of Widespread Mosaicism in Normal Tissues

These very low methylation levels were first validated on another DNA extracted from the same blood sample (except for patient #8 as the first sample was DNA that had been transmitted to the lab). They also had to be confirmed on a second blood sample (mandatory in clinical settings). Highly sensitive techniques such as ddMSP could detect contaminating circulating tumor DNA (ctDNA) within plasma when DNA is extracted from whole blood, especially during active phases of tumor growth or of chemotherapeutic treatment. This might be the case mostly for patients #7 and #8, whose first blood sample was collected either during the first round of chemotherapy or at an advanced stage of the disease. For the five other patients, the first blood sample was collected at a time of controlled disease distant from chemotherapeutic treatment, if any. Absence of significant amount of circulating DNA in the samples was checked using the cell‐free DNA ScreenTape assay, and was validated for the first blood‐extracted DNA samples of the seven patients (Figure S5). Particular attention was also given to ensure that the second blood sample was collected at a time distant from cancer diagnosis, when the patients were considered in remission or with complete response to immunotherapy, to make the detection of methylated ctDNA very unlikely. For patient #8, a third blood sample was also collected, 4 years after the first sample, with complete response to pembrolizumab treatment. For patient #72, the second blood sample could not be performed.

**Figure 4:**
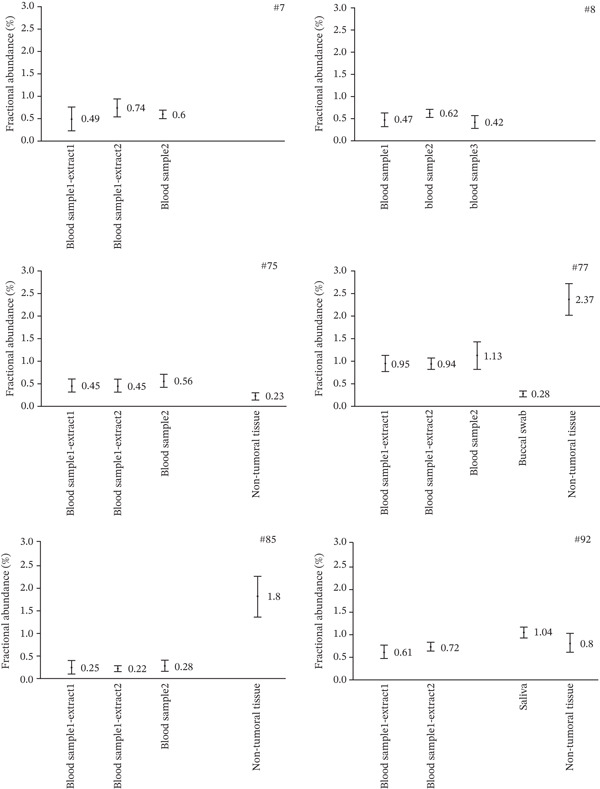
Validation of the very low *MLH1* methylation level in DNA extracted from normal tissues of different embryonic layer origins. *MLH1* methylation levels assessed by ddMSP in DNA extracted from blood (first sample, first and second extractions, and second sample), buccal swab or saliva, and non‐tumoral FFPE tissue distant from the tumor, of the six patients with very low constitutional methylation. Fractional abundance, corresponding to the methylation level, was determined for each sample using Poisson statistical distribution law, with error bars indicating the minimum and maximum values as calculated with Poisson law.

Other sources of germline DNA were also used to validate the constitutional origin of methylation. Saliva was provided by one patient (#92), and buccal swab by two other patients (#75 and #77). Non‐tumoral FFPE tissue distant from the tumor was available for four patients (#75, #77, #85, and #92). These non‐tumoral tissues all came from tumor surgical removal (colectomy or hysterectomy, paraffin block labeled as non‐tumoral), except one. For patient #92, it was an appendix sample removed during distant appendectomy due to appendicitis. *MLH1* methylation was confirmed by ddMSP in all those samples, except the buccal swab from patient #75 for which amplification failed with only 10 DNA molecules analyzed (although performed in duplicate). Comparable low levels of methylation were detected (Figure 4 and Table S2), below 1% in most blood samples, saliva or buccal swab and non‐tumoral tissues, and slightly higher in the tissue from which the tumor originated for two patients (#77 and #85). *MLH1* methylation analysis on available samples revealed widespread mosaicism in normal tissues, thus validating the interest of highly sensitive ddMSP for the diagnosis of constitutional epimutations. For one patient (#72), no control sample was available for validation, so this patient was excluded from further analyses.

### 3.3. Tumoral Analysis

**Figure 5:**
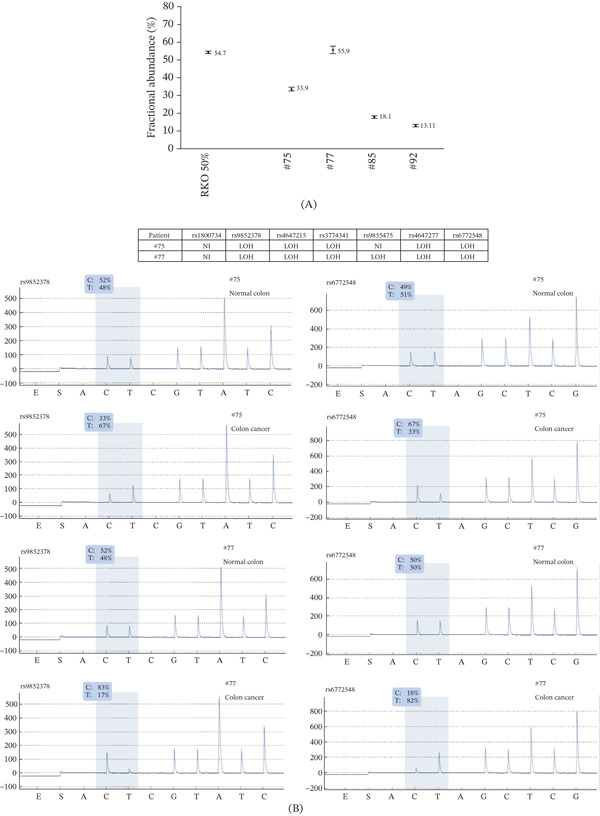
*MLH1* analysis in tumors. (A) *MLH1* methylation levels assessed by ddMSP in bisulfite‐converted DNA from the *MLH1*‐methylated RKO CRC cell line diluted to 50% methylation with DNA from the *MLH1*‐unmethylated HCT116 CRC cell line (RKO 50%) (pooled results from triplicates with independent bisulfite conversion) and from the colon adenocarcinoma of patient #75 (40%–50% MSI cells in the sample), the colon adenocarcinoma of patient #77 (60%–70% MSI cells), the endometrial adenocarcinoma of patient #85 (20%–30% MSI cells), and the duodenal adenocarcinoma of patient #92 (30%–40% MSI cells). Fractional abundance, corresponding to the methylation level, was determined for each sample using Poisson statistical distribution law, with error bars indicating the minimum and maximum values as calculated with Poisson law. (B) Loss of heterozygosity (LOH) detection in the tumors of patients #75 and #77. Representative pyrosequencing traces of informative SNP showing LOH are presented for patients #75 and #77. NI, non‐informative SNP due to homozygous genotype.

*MLH1* methylation level assessed by ddMSP was 33.9% in the colon adenocarcinoma of patient #75 (40%–50% MSI cells in the sample), 55.9% in the colon adenocarcinoma of patient #77 (60%–70% MSI cells), 18.1% in the endometrial adenocarcinoma of patient #85 (20%–30% MSI cells) and 13.11% in the duodenal adenocarcinoma of patient #92 (30%–40% MSI cells) (Figure 5A and Table S2). Tumors from patients #75 and #77 showed LOH (Figure 5B). LOH could not be accurately assessed for patient #92 because informative SNP were located within the duplicated region.

As part of the clinical care procedure, an analysis of the MMR genes was performed in the tumor of patients #8 and #85. A somatic pathogenic variant of the *MLH1* gene was detected at an allelic ratio of 16% in patient #85 endometrial tumor (NM_000249.4:c.2044del, p.(Met682Cysfs ^∗^101)). There was no LOH in this tumor. A *MLH1* somatic pathogenic variant was also detected in the colon adenocarcinoma of patient #8 (NM_000249.4: c.1489del, p.(Arg497Glyfs ^∗^11)).

Overall, an inactivating mechanism of the *MLH1* gene (either LOH or a pathogenic variant) was identified for 4 of the 5 tumors that were analyzed. This argues against biallelic hypermethylation in these tumors (hallmark of sporadic tumors). The additional inactivating mechanism is most likely the second somatic hit leading to MLH1 deficiency, with monoallelic (constitutional) methylation being the first hit.

### 3.4. Frequency of *MLH1* Constitutional Epimutations Among Patients With a *MLH1*‐Methylated Tumor Diagnosed Before Age 61

In this study, we identified six patients with confirmed mosaic constitutional methylation (after exclusion of the patient for whom validation data is missing) in a consecutive series of 142 patients (143‐1) with a dMMR *MLH1*‐methylated tumor diagnosed ≤ 60 years old. This represents 4.2% of the patients (6/142) (7%, if only patients ≤ 55 years old. are considered). Over the same period, using the same selection criteria (*MLH1*‐methylated tumor ≤ 60 years old), 15 patients were identified as *MLH1* epimutation carriers with the pyrosequencing assay we have been using until now for diagnosis. Taking into account the three patients for whom reanalysis with ddPCR was not possible due to lack of remaining DNA sample, the total number of patients is 160 (142 + 15 + 3), with 21 (6 + 15) patients identified as epimutation carriers, which represents 13.1% of the patients with a *MLH1*‐methylated tumor diagnosed before 61. The frequency increases if only patients below 56 are considered (19.6%, 20/102), but that implies missing one diagnosis.

### 3.5. *MLH1* Constitutional Variant Screening in Patients With Very Low *MLH1* Methylation Level

For the six newly diagnosed patients, we sequenced the 5 ^′^ part of the *MLH1* gene (5 ^′^UTR, exon 1 and the 200 first bp of intron 1) to search for *MLH1* variants previously reported as associated with low‐level constitutional methylation (i.e., NM_000249.4: c.27G>A and c.116+106G>A) [16, 24]. Those variants were not detected. The c.‐27C>A variant [12] was also not detected.

As part of the initial diagnosis procedure, germline genetic testing of the MMR genes was performed for all the patients except patient #77, either as the first‐line molecular testing or after the negative *MLH1* epimutation screening result, due to young age at diagnosis (to exclude methylation as the second hit with a germline pathogenic variant as the first hit). No germline pathogenic or probably pathogenic variant was identified in the *MLH1* gene. Only a heterozygous duplication of *MLH1* exons 1–13, considered as a variant of unknown significance, was identified for patient #92. As no relative has been tested yet and no further analysis performed, the link between this duplication and promoter methylation has not been confirmed.

### 3.6. *MLH1* Epimutation Screening in Relatives

Only three first‐degree relatives could be tested (two from patients #8 and one from patient #75, Table 1). *MLH1* methylation was not detected in DNA extracted from their blood. Patient #75′s older sister developed a colon cancer at the age of 50 but this tumor was pMMR (MMR proficient) with retained expression of the four MMR proteins, consistent with absence of significant methylation detected by ddMSP (0.014%, min–max [0–0.048]).

### 3.7. ddMSP for the Validation of Low‐Level Methylation Detected by Pyrosequencing

**Figure 6:**
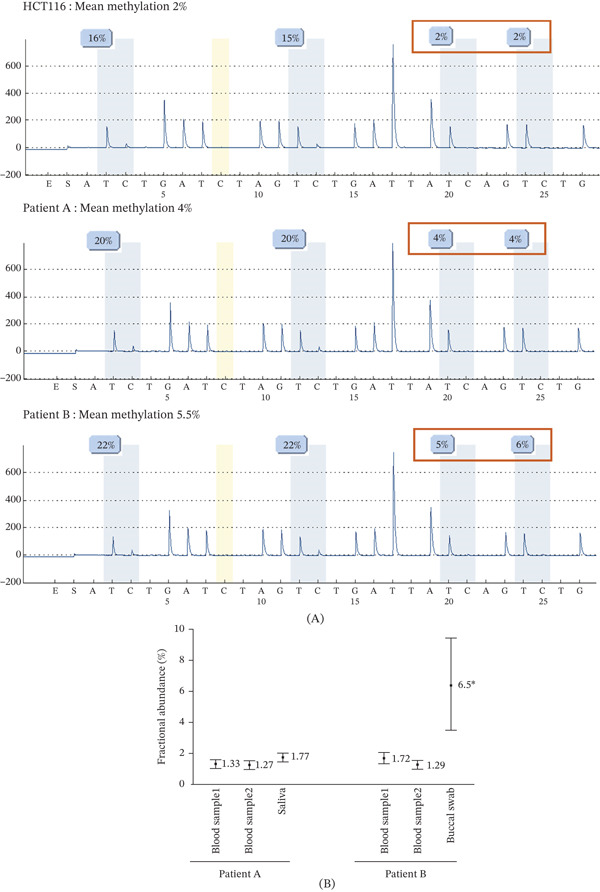
Validation of low‐level *MLH1* constitutional methylation with ddMSP: comparative results from pyrosequencing and ddMSP. (A) *MLH1* methylation levels assessed by pyrosequencing in bisulfite‐converted DNA from blood Sample 1 of patients A and B and from the HCT116 negative control from the same experiment. The mean methylation level is calculated using the last two CpG sites (red rectangle). (B) *MLH1* methylation levels assessed by ddMSP in bisulfite‐converted DNA from blood Samples 1 and 2, and from saliva or buccal swab, of patients A and B. Fractional abundance, corresponding to the methylation level, was determined for each sample using Poisson statistical distribution law, with error bars indicating the minimum and maximum values as calculated with Poisson law. ∗Unreliable quantification (but reliable positive result) due to the very low number of DNA molecules screened (370) in buccal swab from Patient B (although sample run in duplicate).

During the implementation phase of the ddMSP technique in the clinical lab, ddMSP was also useful for some patients with low levels of methylation (Figure 6). The first patient (Patient A) was a male who presented with a proximal colon adenocarcinoma at the age of 50, with loss of MLH1 and PMS2 expression by IHC and *MLH1* methylation. The second patient (Patient B) was a female who developed a proximal colon adenocarcinoma (pT3N2) at the age of 49 and an endometrioid adenocarcinoma of the ovary at the age of 50. Both tumors showed MSI, IHC loss of MLH1 and PMS2 expression and *MLH1* methylation. For these two patients, *MLH1* methylation levels detected by pyrosequencing in DNA extracted from blood were above the LOD (limit of detection) of our pyrosequencing assay (i.e., 3.7%), with 4% for Patient A and 5.5% for Patient B (Figure 6A). ddMSP was used to validate the low‐level methylation on the same DNA sample (1.33% for Patient A and 1.72% for Patient B), and to further confirm the constitutional epimutation for these patients, in DNA extracted from another blood sample and from saliva or buccal swab (Figure 6B and Table S2). *MLH1* methylation level was also assessed by ddMSP in the colon adenocarcinoma of Patient B (50%–60% MSI cells in the sample), and was 46.6%. LOH was detected in this tumor, and explained why the percentage of methylation and of MSI cells were quite similar even though methylation is monoallelic.

## 4. Discussion

Variable levels of methylation are observed in the blood of *MLH1* epimutation carriers, ranging from low‐level (1%–5%) mosaic methylation to hemiallelic methylation (~50%) [16–20, 22]. In this study, we show that methylation level can be extremely low (below 1%) in some patients, and that these patients can be missed if highly sensitive assays are not used for epimutation screening.

One hundred forty‐three patients with a *MLH1*‐methylated dMMR tumor before 61 years old who had been tested in our clinical lab between 2020 and 2024 and considered as non‐carrier after *MLH1* constitutional epimutation screening with the pyrosequencing assay were re‐tested using ddMSP. With this highly sensitive assay, a methylation level below 1% was detected for seven patients. DNA has been extracted from whole blood and such very low levels of methylation raise the question of potential detection of methylated plasma‐ctDNA as tumors from these patients show *MLH1* promoter methylation. To rule out this hypothesis, several validation steps were performed. First, another DNA specimen was extracted from the same blood sample, which had been frozen and stored in the lab, and methylation was detected at similar level in these new specimens. Second, *MLH1* methylation was further validated on a new blood sample that was collected several months after primary tumor management, when the patients were considered in remission. Although residual disease is still possible for a few patients, this makes the detection of methylated ctDNA unlikely. Third, other available sources of germline DNA (saliva, buccal swab, and non‐tumoral tissue distant from the tumor) were tested to validate the constitutional origin of methylation and confirmed soma‐wide mosaicism. Fourth, tumor DNA analysis was performed to identify a second inactivating somatic hit in the *MLH1* gene (LOH or pathogenic variant) indicative of monoallelic methylation which is an hallmark of constitutional epimutations whereas somatic methylation observed in sporadic tumors is biallelic. As these validation steps are essential to confirm the constitutional origin of methylation, we excluded one of the seven patients from frequency calculation, because no control sample was available to validate the very low methylation level.

This study highlights the critical importance of highly sensitive assays for the identification of epimutation carriers. We chose ddMSP among other robust and highly sensitive assays because of its increased sensitivity and of absolute quantification based on the direct count of positive partitions [25]. By partitioning the sample into thousands of individual reactions, dPCR increases the sensitivity of target detection, even at very low concentrations and allows the detection of rare methylated targets in a background of non‐methylated DNA. As PCR occurs in isolated partitions, the impact of inhibitors is reduced. In addition, dPCR enables the precise determination of the total number of DNA copies analyzed in a sample, which prevents the risk of false negatives due to an insufficient number of copies analyzed. With a minimum of 3000 copies of DNA analyzed per blood sample, samples with methylation level ≥ 0.1% cannot be missed.

Our ddMSP assay enabled the identification of additional patients who will benefit from LS cancer surveillance programs. These patients were first considered, after initial epimutation and genetic testing, as having a possible sporadic tumor, even if clinical surveillance was maintained for the youngest patients. Identification of a constitutional epimutation allows conclusive diagnosis of LS for these patients. Moreover, presymptomatic testing becomes possible for relatives. A *MLH1* genetic variant was only detected for one patient (i.e., a duplication of exons 1–13), suggesting a potential secondary epimutation. In this case, epimutation testing in relatives is highly relevant and will help to determine if the epimutation is located on the duplicated allele. For the other patients, the lack of family history and the lack of genetic variant identified in the *MLH1* gene point towards *de novo* primary epimutations. However, highly sensitive epimutation testing could still be proposed to first‐degree relatives as a *MLH1* genetic alteration could have been missed and disease penetrance in LS is incomplete.

Several patients with low‐level *MLH1* constitutional methylation have been reported as having multiple LS‐associated tumors and/or young age of tumor onset [16, 18, 19], indicating that even low‐level mosaic methylation can confer a high risk of LS‐associated tumors. We restricted our study to patients below 61 years old. and consequently could not identify epimutation carriers above this age. However for more than half (4/7) of the patients we identified with a methylation level below 1%, age of tumor onset was ≤ 45 years old, supporting previous findings that the degree of blood‐based methylation does not correlate with cancer risk.

*MLH1*‐methylated tumors are still too often considered as sporadic tumors, leading to exclusion of the patients from genetic counseling and germline testing [1]. This study highlights the importance of constitutional epimutation screening for patients with a dMMR *MLH1*‐methylated tumor before age 61, as 13.1% of the patients were identified as epimutation carriers. The large number of patients included in this study enabled us to estimate the frequency of epimutation carriers in that group of patients with good confidence. This data and the increasing number of epimutation carriers reported in the literature should help constitutional epimutation screening become a standard of care for patients with a *MLH1*‐methylated tumor. Most *MLH1* constitutional epimutations are primary epimutations, which arise *de novo*, with no family history. Consequently, family history cannot be integrated as a major criterion in the decision algorithm for methylation testing, and the age of onset of the *MLH1*‐methylated tumor is the main criterion for patients with a single tumor. However, the tumor‐onset age limit for optimal systematic *MLH1* constitutional epimutation screening remains an open question. Indeed, as previously reported [20], diagnosis yield increases if epimutation screening is restricted to patients with a *MLH1*‐methylated tumor before age 56 (it reaches 19.6% in this study). However, as otherwise reported [19], it also means missing a few patients. Consequently, we recommend that every patient with a *MLH1*‐methylated tumor diagnosed before age 61 benefit from constitutional epimutation screening. Additionally, in the context of the increasing assessment of MMR status in tumors (particularly for guiding treatment with immune checkpoint inhibitors) and of the increasing number of *MLH1*‐methylated tumors that will be identified as the age distribution advances, it should be kept in mind that constitutional epimutation screening may also be clinically relevant for some patients above 61, including those with multiple *MLH1*‐methylated tumors.

In conclusion, *MLH1* constitutional epimutation screening should become a standard of care for patients with a *MLH1*‐methylated tumor before 61. Optimal screening requires highly sensitive assays to identify patients with very low mosaic methylation levels. Validation of these very low levels on another blood sample collected at distance from tumor management and on samples originating from other embryonic layers remains mandatory to confirm the constitutional epimutation for diagnostic purposes.

## Author Contributions

Cédric Facon and Catherine Vermaut contributed equally to this work.

## Funding

This study was supported Ligue Contre le Cancer Septentrion Commitee, 2022 Canceropole Nord‐Ouest; Inserm n° 2021/11, emerging projects 2021 Inserm‐Contrat d′interface pour hospitaliers.

## Conflicts of Interest

The authors declare that there is no conflict of interest regarding the publication of this article.

## Supporting Information

Additional supporting information can be found online in the Supporting Information section.

## Supporting information


**Supporting Information 1** Figure S1: *MLH1* promoter methylation analysis by ddMSP: specificity of the assays for the amplification and detection of methylated DNA templates (M‐assay) or unmethylated DNA templates (UM‐assay).

**Supporting Information 2** Figure S2: Linearity of *MLH1* methylation detection using ddMSP.

**Supporting Information 3** Figure S3: Fractional abundance values as a function of the number of DNA molecules screened for RKO 0.1% samples.

**Supporting Information 4** Figure S4: Distribution by age of the patients, according to the type of tumors (digestive cancers or gynecological cancers).

**Supporting Information 5** Figure S5: Absence of significant amount of circulating DNA in DNA samples extracted from blood of the patients with very low methylation levels.

**Supporting Information 6** Table S1: Primer and probe sequences. Table S2: Methylation levels assessed by ddMSP in the different DNA samples of the patients with *MLH1* promoter methylation identified in the study.

## Data Availability

The data that support the findings of this study are available on request from the corresponding author. The data are not publicly available due to privacy or ethical restrictions.
