# Supplementary material for: MLH1 Constitutional Epimutation Screening Requires Highly Sensitive Assays to Identify Lynch Syndrome Patients With Very Low Mosaic Methylation Level
Source: Hum Mutat. 2026 May 14;2026:6909313. doi: 10.1155/humu/6909313 (PMC13173757; doi:10.1155/humu/6909313)
Supplement: Supplementary file 1 — Supporting Information 1 Figure S1: MLH1 promoter methylation analysis by ddMSP: specificity of the assays for the amplification and detection of methylated DNA templates (M‐assay) or unmethylated DNA templates (UM‐assay). [file HUMU-2026-6909313-s002.pptx]

## Slide 1
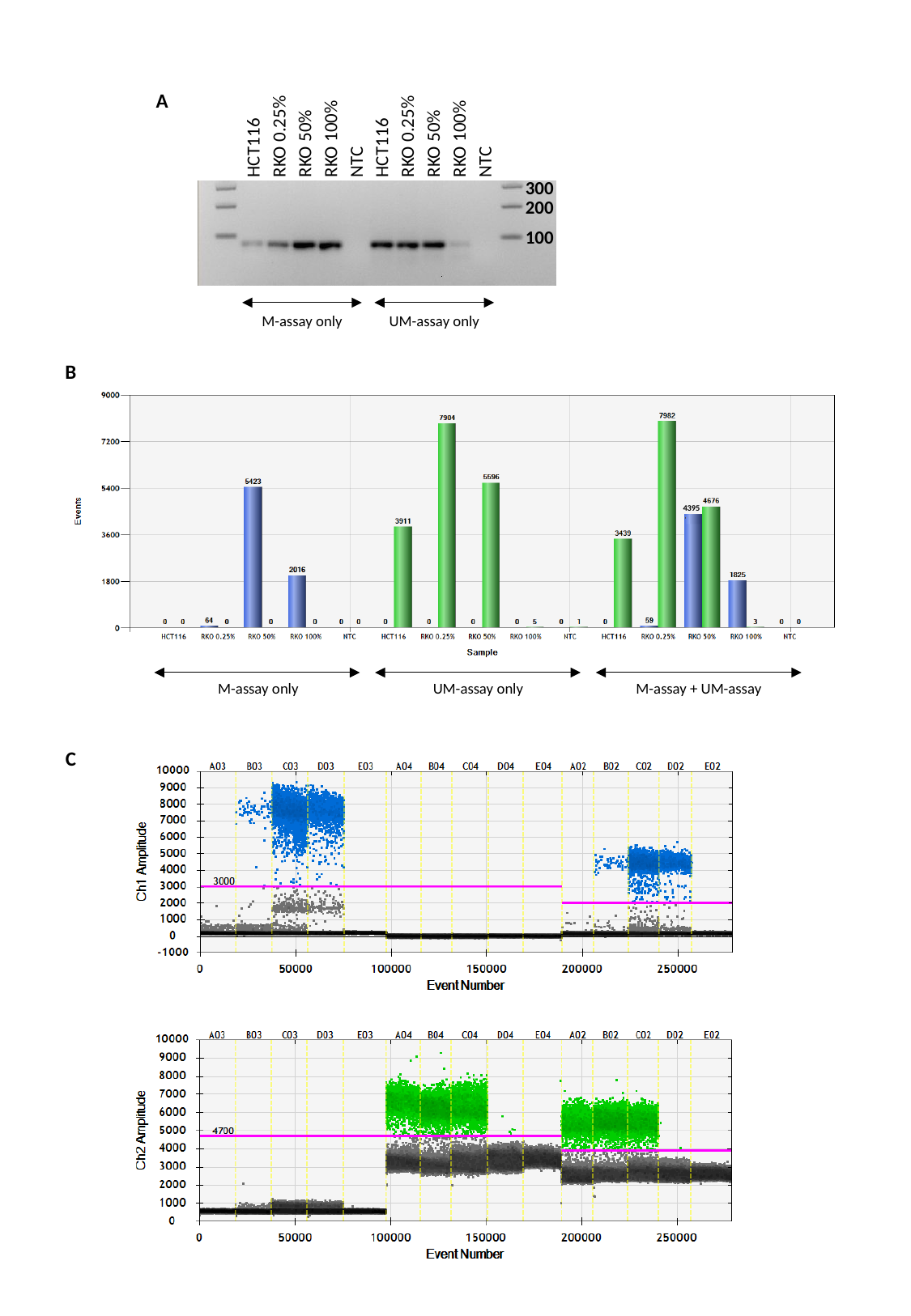

HCT116
RKO 0.25%
RKO 50%
RKO 100%
NTC
HCT116
RKO 0.25%
RKO 50%
RKO 100%
NTC
300
200
100
M-assay only
UM-assay only
A
B
4395
M-assay only
UM-assay only
M-assay + UM-assay
C
