# Supplementary figures and images for: MLH1 Constitutional Epimutation Screening Requires Highly Sensitive Assays to Identify Lynch Syndrome Patients With Very Low Mosaic Methylation Level
Source: Hum Mutat. 2026 May 14;2026:6909313. doi: 10.1155/humu/6909313 (PMC13173757; doi:10.1155/humu/6909313)

## Slide 1
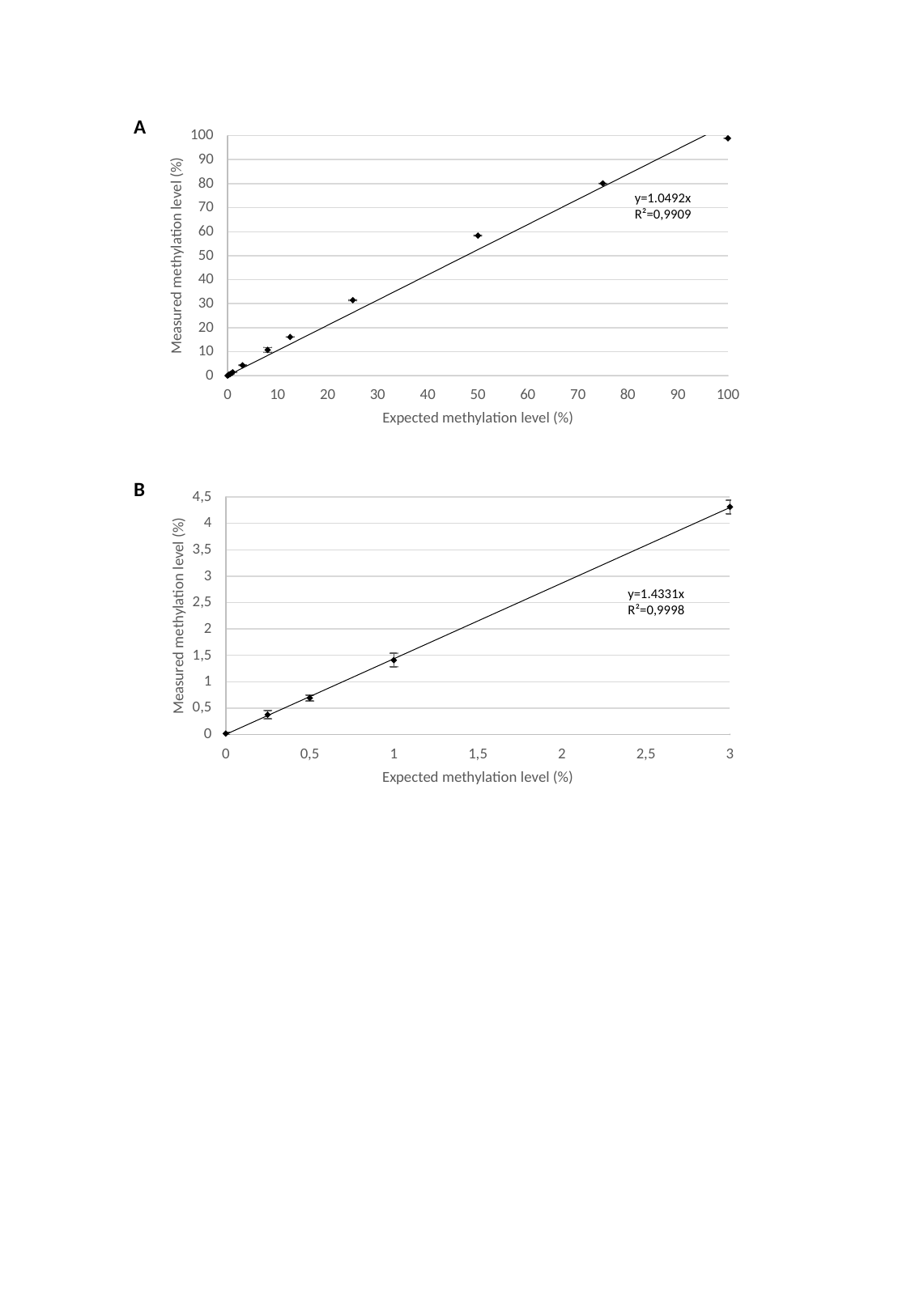

A
y=1.0492x
R²=0,9909
B
y=1.4331x
R²=0,9998

Supplement: Supplementary file 2 — Supporting Information 2 Figure S2: Linearity of MLH1 methylation detection using ddMSP. [file HUMU-2026-6909313-s003.pptx]

## Slide 1
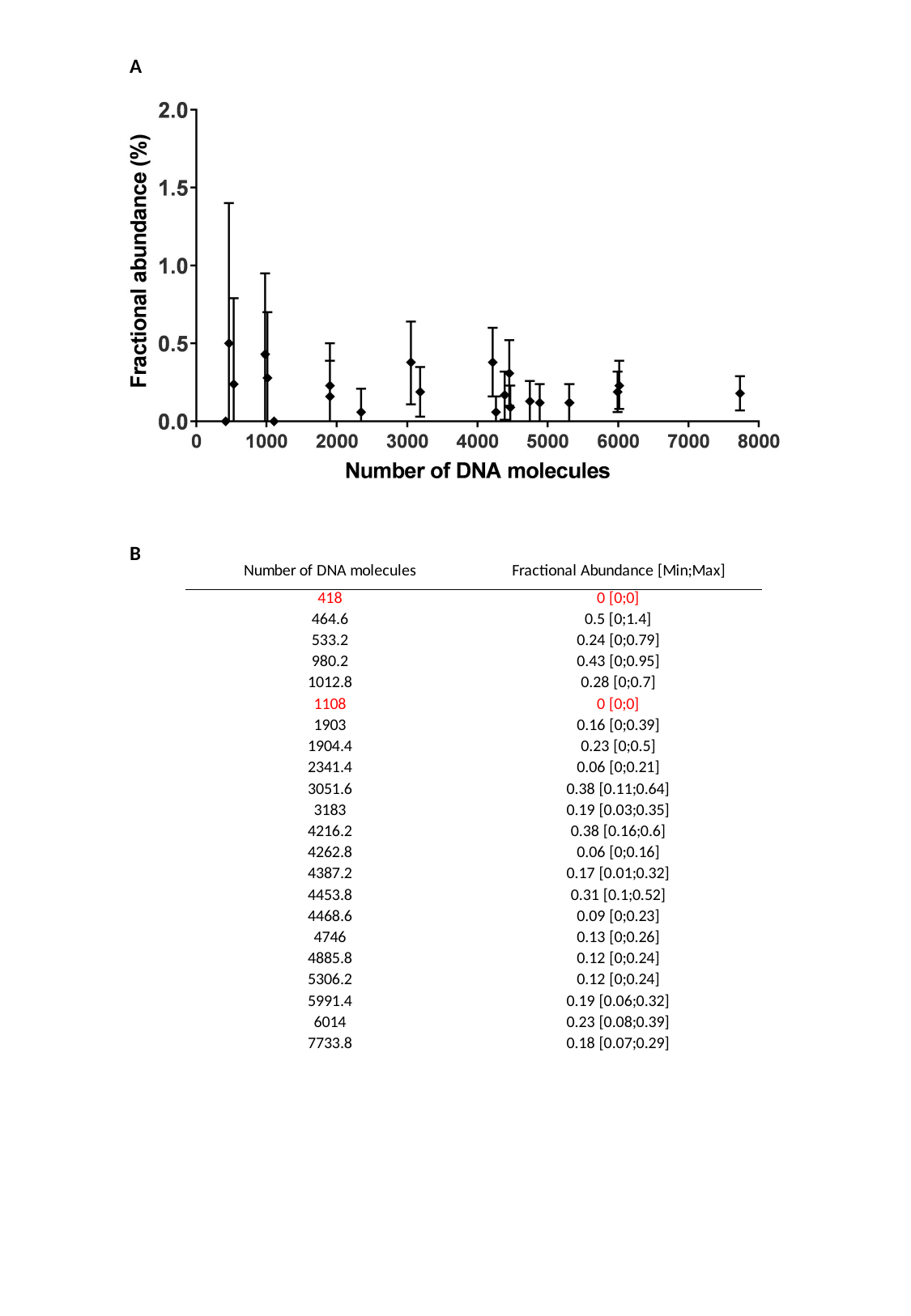

A
B

Supplement: Supplementary file 3 — Supporting Information 3 Figure S3: Fractional abundance values as a function of the number of DNA molecules screened for RKO 0.1% samples. [file HUMU-2026-6909313-s004.pptx]

## Slide 1
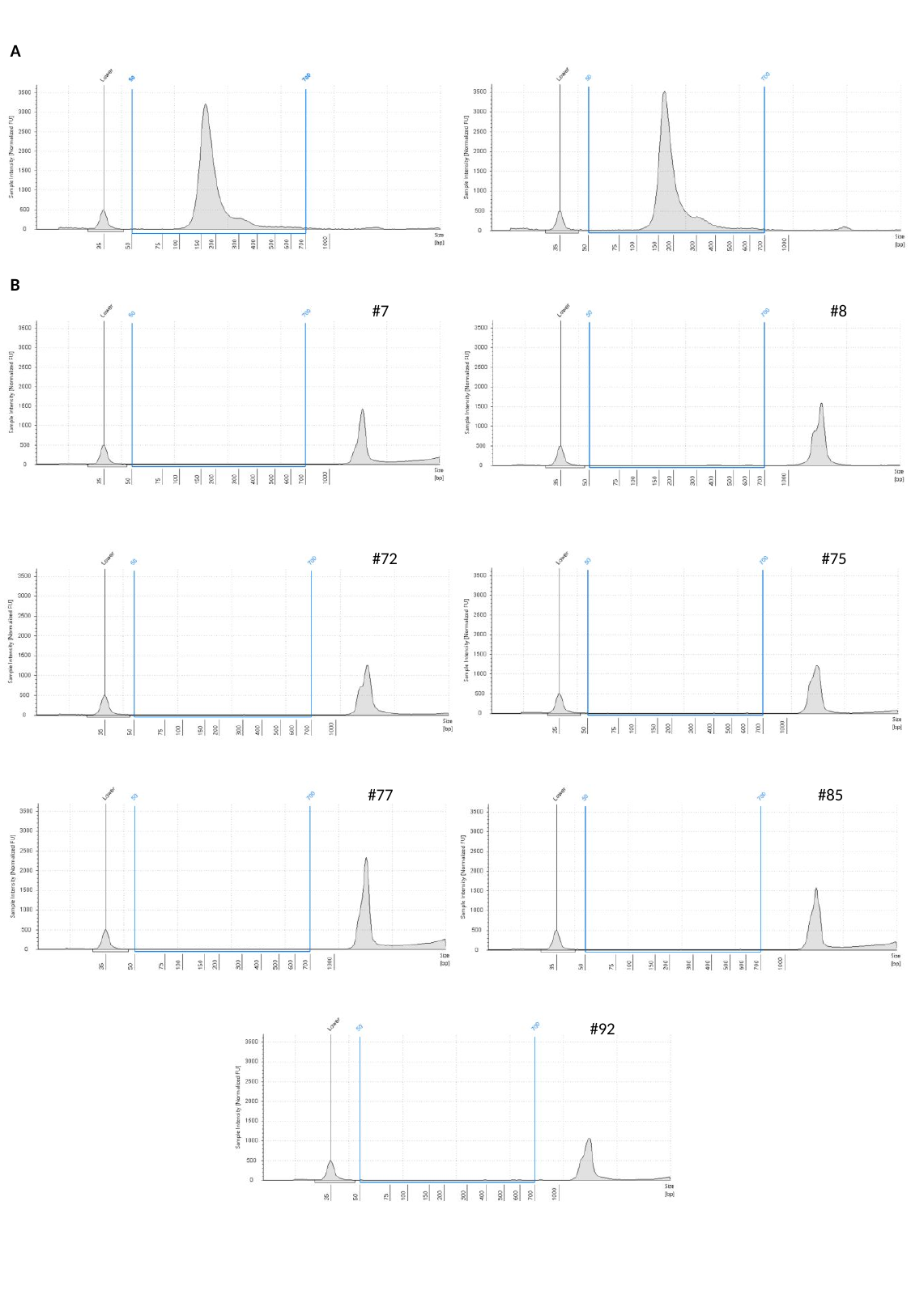

A
B
#8
#7
#75
#72
#77
#85
#92

Supplement: Supplementary file 5 — Supporting Information 5 Figure S5: Absence of significant amount of circulating DNA in DNA samples extracted from blood of the patients with very low methylation levels. [file HUMU-2026-6909313-s005.pptx]
