# Supplementary material for: MLH1 Constitutional Epimutation Screening Requires Highly Sensitive Assays to Identify Lynch Syndrome Patients With Very Low Mosaic Methylation Level
Source: Hum Mutat. 2026 May 14;2026:6909313. doi: 10.1155/humu/6909313 (PMC13173757; doi:10.1155/humu/6909313)
Supplement: Supplementary file 4 — Supporting Information 4 Figure S4: Distribution by age of the patients, according to the type of tumors (digestive cancers or gynecological cancers). [file HUMU-2026-6909313-s001.docx]

**A**

18

34

1

2

1

2*

4

5

7

12

12

**B**

5

3

1

7

14

22
