# Supplementary material for: MLH1 Constitutional Epimutation Screening Requires Highly Sensitive Assays to Identify Lynch Syndrome Patients With Very Low Mosaic Methylation Level
Source: Hum Mutat. 2026 May 14;2026:6909313. doi: 10.1155/humu/6909313 (PMC13173757; doi:10.1155/humu/6909313)
Supplement: Supplementary file 6 — Supporting Information 6 Table S1: Primer and probe sequences. Table S2: Methylation levels assessed by ddMSP in the different DNA samples of the patients with MLH1 promoter methylation identified in the study. [file HUMU-2026-6909313-s006.docx]

**Supplementary material**

**Supplemental Figure 1. *MLH1* promoter methylation analysis by ddMSP: specificity of the assays for the amplification and detection of methylated DNA templates (M-assay) or unmethylated DNA templates (UM-assay).** Analysis was performed in single wells with only the M-assay or the UM-assay, and in wells with both assays. Bisulfite-treated DNA from the *MLH1*-unmethylated HCT116 CRC cell line (HCT116), the *MLH1*-methylated RKO CRC cell line (RKO 100%), and RKO diluted to 0.25% methylation (RKO 0.25%) or to 50% methylation (RKO 50%) with DNA from HCT116, and a no-template control (NTC) were analyzed. **(A)** Gel electrophoresis after PCR amplification within the droplets. A faint band is observed for HCT116 with the M-assay and for RKO with the UM-assay, indicating that specificity is not fully achieved with primers only. **(B)** Specificity achieved by primers + probes (event charts from QuantaSoft Analysis software, BioRad). The number of events is indicated above each histogram bar (left blue bar: FAM-positive, right green bar: HEX-positive). The absence of event detected as HEX-positive with the M-assay only, and the absence of event detected as FAM-positive with the UM-assay only show the absence of aspecific fluorescent signal. The absence of FAM-positive event for HCT116 show the specificity of the M-assay. The extremely low number of HEX-positive events for RKO show the specificity of the UM-assay, as a very small proportion of unmethylated molecules in this cell line cannot be excluded. **(C)** Fluorescence intensity of the droplets (1D amplitude charts from QuantaSoft Analysis software). **Up**, FAM-positive droplets (blue) correspond to amplification of methylated DNA. Grey droplets correspond to empty droplets (no amplification). The intensity threshold set for fluorescent signal is indicated by a pink line. The samples are in the same order as in Figure B (wells A: HCT116, B: RKO 0.25%, C: RKO 50%, D: RKO 100%, E: NTC; wells 03: M-assay only, 04: UM-assay only, 02: M-assay + UM-assay). **Down**, HEX-positive droplets (green) correspond to amplification of unmethylated DNA.

**Supplemental Figure 2. Linearity of *MLH1* methylation detection using ddMSP.** Measured versus expected methylation levels from a dilution series of the *MLH1*-methylated RKO CRC cell line with the *MLH1*-unmethylated HCT116 CRC cell line are presented. The mean methylation level from two independent experiments (each with measure in triplicate) is shown for each dilution point, with errors bars corresponding to standard deviation. **(A)** Full range of values from 0% to 100%, with strong linear correlation (R²=0.9909). **(B)** Lower range of values, with strong linear correlation (R²=0.9998).

**Supplemental Figure 3. Importance of the number of DNA molecules screened by ddMSP for samples with a very low methylation level: results from 22 RKO 0.1% samples. (A)** Fractional abundance values presented as a function of the number of DNA molecules screened for 22 RKO 0.1% samples (variable DNA input). **(B)** Table with data for each RKO 0.1% sample. In red, 2 samples with failed detection of methylated molecules (fractional abundance = 0) due to insufficient number of molecules screened (418 and 1108 molecules respectively).

**Supplemental Figure 4.** **Histograms showing the distribution by age range of the patients included in the study, according to the type of tumors.** **(A)** Patients with a digestive cancer. **(B)** Patients with a gynecological cancer. Patients with very low constitutional *MLH1* methylation (in orange, numbers indicated on the right of the histogram bars) detected among cancer cases with a dMMR *MLH1*-methylated tumor diagnosed ≤60 y.o. who were previously considered as non epimutation-carriers with pyrosequencing (in grey, numbers indicated above the histogram bars). Five-year age bins are represented. *as no control sample was available for validation, one patient was excluded from further analyses.

**Supplemental Figure 5. Absence of significant amount of circulating DNA in DNA samples extracted from blood of the patients with very low methylation levels.** The cell-free DNA ScreenTape assay and the 4200 TapeStation system (Agilent) were used to assess the presence of cell-free DNA (50–800 bp in size). **(A)** Two circulating DNA specimens were run as positive controls. They correspond to 98% and 97% cell-free DNA, and show a characteristic peak at 200 bp. **(B)** Absence of the 200 bp peak for DNA samples extracted from blood of the 7 patients (#7, #8, #72, #75, #77, #85 and #92), indicating the absence of small DNA molecules corresponding to circulating DNA.

**Supplemental Table 1.**

| Application | Sequences (5’->3’) | Amplicon size (bp) |
| --- | --- | --- |
| ddMSP M-assay | F: GCGGATAGCGATTTTTAACGC * | 76 |
|  | R: CTTCGTCCCTCCCTAAAACGA * |  |
|  | P: AGCGTATATTTTTTTAGGTAGCG * |  |
| ddMSP UM-assay | F: GTGGATAGTGATTTTTAATGT | 76 |
|  | R: CTTCATCCCTCCCTAAAACAA |  |
|  | P: AGTGTATATTTTTTTAGGTAGTG |  |
| Sanger sequencing : MLH1 exon 1 | F: GGCAGTAGCCGCTTCAGGGA | 553 |
|  | R: CTTAAAAGGTCTCGGGGGAG |  |

**Supplemental Table 1. Primer and probe sequences.** *, from Pinto et al. [22]

**Supplemental Table 2.**

**Supplemental Table 2. Methylation levels assessed by ddMSP in the different DNA samples of the patients with *MLH1* promoter methylation identified in the study.** Fractional abundance with minimum and maximum values under brackets, as calculated with Poisson statistical distribution law.

*unreliable quantification value due to the very low number of DNA molecules screened in buccal swab from patient B (high minimum-maximum range).
